# The prevalence of elder abuse and risk factors: a cross-sectional study of community older adults

**DOI:** 10.1186/s12877-023-04307-0

**Published:** 2023-09-30

**Authors:** Reza Nemati-Vakilabad, Zahra Khalili, Leila Ghanbari-Afra, Alireza Mirzaei

**Affiliations:** 1 School of Nursing and Midwifery, Guilan University of Medical Sciences, Rasht, 141973317 Iran; 2 Department of Medical-Surgical Nursing, School of Nursing and Midwifery, Ardabil University of Medical Sciences, Ardabil, Iran; 3 Trauma Nursing Research Center, Kashan University of Medical Sciences, Kashan, Iran; 4 Department of Emergency Nursing, School of Nursing and Midwifery, Ardabil University of Medical Sciences, Ardabil, Iran

**Keywords:** Elder abuse, Risk factors, Iran

## Abstract

**Background:**

The old people population is increasing worldwide. Along with their increasing population, an increase in elder abuse cases is predicted. Elder abuse is a neglected problem, and many cases go unreported. This study was conducted to identify types of elder abuse and examine associated risk factors.

**Methods:**

This cross-sectional analytical study was conducted on 500 older people in Ardabil (northwestern Iran). Data was collected over three months, from June to September 2020. Data was collected using a demographic information form and the Domestic Elder Abuse questionnaire. The data were analyzed using SPSS software (version 22). Logistic regression was used to identify factors related to elder abuse.

**Results:**

The results showed that out of the 500 participants, 258 (51.6%) were male, and 242 (48.2%) were female. Among the 500 participants, 377 individuals (75/4%) reported experiencing at least one type of abuse in the past year. The highest rate of elder abuse was observed for emotional neglect (47.2%) and psychological abuse (40.8%), while the lowest rate was measured for rejection (15.4%) and physical abuse (12.4%). The results indicated that elder abuse was significantly associated with chronic illness (OR = 0.601, 95% CI: 0.391–0.922) and having 1–4 children (OR = 1.275, 95% CI: 1.137–1.430).

**Conclusion:**

Considering the high level of elder abuse and its dangerous effects on the quality of life for older people, it is essential to develop appropriate programs to increase awareness among older people and their families.

## Introduction

One of the challenges of the 21st century is the increasing number of older people in developing countries [1]. According to the World Health Organization (WHO), the number of people over 60 is expected to double by 2050, requiring significant social changes [2]. Iran is undergoing a critical transition toward an aging society [3]. Based on the latest census in 2014, individuals aged 60 and above make up 9.2% of Iran’s total population [4]. Therefore, it is estimated that the number of older people in Iran will reach approximately 21–25% by 2051 [3].

On the other hand, it is predicted that with the increase in the global older people population, the number of older people experiencing abuse will also increase [5]. Elder abuse is a global public health and human rights problem [6]. Therefore, the WHO defines elder abuse as “a behavior that intentionally or unintentionally reduces the functioning of older adults and increases their physical and psychological harm“ [7]. This abuse includes physical, sexual, psychological, economic, and neglect. Since elder abuse is a stressful event, it can have various dimensions, such as emotional neglect, care neglect, financial neglect, financial abuse, rejection, psychological abuse, physical abuse, abandonment, and sexual abuse [8]. A systematic review and meta-analysis in Iran indicated the impact of all these dimensions on the health of older people [9].

According to the WHO report in 2017, at least one type of abuse is experienced by every six older people aged 60 and above during their lifetime, and their population is exposed to various forms of abuse [10]. Furthermore, a global systematic review and meta-analysis of prevalence studies showed that the overall prevalence of elder abuse in social and educational settings is approximately 14.1% [10]. In East Asian countries, the overall prevalence of elder abuse is 78.33 per 1000 individuals annually [11]. In a systematic review in Iran, the overall prevalence of elder abuse is 48.3%, with the highest majority being neglect and mistreatment [9].

Elder abuse is a complex issue, with various explanations highlighting different factors, such as living conditions or family situations, as well as characteristics of both the victims and the abusers [12]. Studies have shown that several risk factors, such as physical disability, cognitive impairment, dependence on others, poor physical and mental health, low income, lack of social support, and being female, are associated with elder abuse [13]. The adverse effects of abuse extend to mental and physical health, social status, and structures and can even lead to negative consequences such as psychological distress, illness, and death [14]. Furthermore, elder abuse can result in costly outcomes such as hospitalization and relocation to nursing homes [6].

Given the diversity of Iranian culture and its essential role in improving the health and quality of life of older people, as well as the limited studies in this area, conducting research such as the present study is necessary and can help fill the existing gap. Therefore, this study aimed to determine the prevalence of abuse and its associated factors among older adults in Ardabil City.

## Methods

### Setting and participants

The population of this cross-sectional analytical study consisted of older people aged 60 years and older in Ardabil, northwestern Iran, who were selected through cluster sampling. Two healthcare centers were randomly selected from each region (central, northern, southern, western, and eastern). The older people were determined through simple random sampling in each healthcare center based on their family records.

The inclusion criteria were age 60 and above, willingness to participate in the study, no history of hearing impairment, no history of mental illness (self-reported), and ability to answer the questions.

The study population consisted of 500 older people over the age of 60 who had a history of receiving healthcare services at treatment centers in Ardabil. Based on a previous study and an estimated inactivity of 32% among older people, with a confidence level of 95% (d = 0.05, *p* = 0.32, z = 1.96), the sample size was calculated to be 334 according to the following formula [15]. Due to cluster sampling, the sample size was increased by 1.5 times, resulting in a final sample size of 500 individuals for examination (Eq. 1).

1 $$\textrm{n}=\raisebox{1ex}{${\left({\textrm{z}}_{1\frac{\alpha }{2}}\right)}^2\times \textrm{p}\times \textrm{q}$}\!\left/ \!\raisebox{-1ex}{$\textrm{d}2$}\right.$$

### Data collection

The data were collected through interviews with older people in their homes from June to September 2020. These interviews were conducted using a structured interview protocol developed by the researchers and provided by healthcare providers. Phone calls were made to establish an initial agreement for in-home consultations with each selected individual. Then, after explaining the purpose of the study and obtaining informed consent during the visit, participants were privately interviewed.

#### Measure

The data was collected using a demographic information form (including age, gender, education level, number of children, marital status, employment status, residential status, housing type, economic dependency, chronic disease, walking ability, and use of mobility aids) and the Domestic Elder Abuse questionnaire.

##### Domestic elder abuse questionnaire

The Domestic Elder Abuse questionnaire was developed by Heravi-Karimooi et al. [16]. To assess elder abuse in Iranian families that can be used in different situations. The Domestic Elder Abuse questionnaire consists of 49 items divided into eight subscales, including care neglect (11 things), psychological abuse (8 items), physical abuse (4 items), financial abuse (6 items), authority deprivation (10 items), rejection (4 items), economic neglect (4 items), and emotional neglect (2 items). Each item was scored from 1 (none) to 5 (severe). The scale items include the options yes, no, and not applicable. The option “Not applicable” applies when the thing is irrelevant to the respondent’s living conditions. The score range is from zero to 100, and higher scores represent more symptoms of abuse.

The developers of the scale reported the psychometric properties of the tool to be valid in terms of face validity, content validity (CVI, 0.92), construct validity, high internal consistency (Cronbach’s alpha, 0.90 to 0.97), and test-retest reliability (0.99) [17].

### Data analysis

The data were analyzed by SPSS software version 22 (Armonk NY: IBM Crop) using descriptive statistics (percentage and proportion). To investigate the relationship between the prevalence of abuse and demographic variables, the independent-sample t-test and one-way ANOVA were used. Predictors of participants’ prevalence of abuse were determined using logistic regression. The level of significance was considered to be 0.05.

## Result

Of the 500 participants in the present study, 258 (51.6%) were male, and 242 (48.2%) were female. The mean age of the participants was 69.15 ± 7.27 years, with the highest frequency (54.6%) falling within the age range of 60–70 years. Regarding education level, the highest frequency belonged to the primary education group (49.2%), while only 13.2% had received higher education. Most participants (64%) were unemployed and lived independently (70.2%), with 83% owning their own homes. Overall, 45.4% did not have any chronic illnesses. Regarding mobility aids, 21% of older people used no mobility assistance devices. The relationship between demographic characteristics and related factors with elder abuse is shown in Table 1.

**Table 1:** The relationship between demographic and the associated factors the elder abuse (*n* = 500)

Variable	Category	Frequency [%]	Abused	Non-abused	*p*-value
^a^Gender	Female	242 (48.4)	184(76)	58(24)	0.750
	Male	258 (51.6)	193(74.8)	65(25.2)	
^b^Age (year)	60–69	273 (54.6)	202(74)	71(26)	0.712
	70–79	168 (33.6)	130(77.4)	38(22.6)	
	≥ 80	59 (11.8)	45(76.3)	14(23.7)	
^b^Number of children	0	11 (2.2)	7(63.6)	4(36.4)	**0.004**
	1–4	310(62.0)	220(71)	90(29)	
	≥ 5	179(35.8)	150(83.8)	29(16.2)	
^a^Marital status	Married	351(70.2)	265(75.5)	86(24.5)	0.930
	Widowed/single/separated	149 (29.8)	112(75.2)	37(24.8)	
^b^Educational level	Illiterate	188(37.6)	145(77.1)	43(24.8)	0.202
	Primary	246 (49.2)	188(76.4)	58(23.6)	
	Diploma and above	66(13.2)	44(66.7)	22(33.3)	
^b^Living arrangement	Living with spouse	14 (28.2)	112(79.4)	29(20.6)	0.213
	Living with spouse & children	198 (39.6)	140(70.7)	58(29.3)	
	Living with children	101 (20.2)	75(74.3)	26(25.7)	
	Living alone	60 (12.0)	50(83.3)	10(16.7)	
^b^Employment status	Unemployed	257 (78.2)	201(78.2)	56(21.8)	0.325
	Employed	86 (17.2)	62(72.1)	24(27.9)	
	Pensioner	157 (31.4)	114(72.6)	43(27.4)	
^b^Residential status	Leased	42 (8.4)	39(92.9)	3(7.1)	**0.004**
	Landlord	415(83.0)	300(72.3)	115(27.7)	
	Children’s home	43 (8.6)	38(87.5)	5(12.5)	
^b^Housing type	Apartment	88 (17.6)	73(83)	15(17)	**0.041**
	Home without a yard	148(29.6)	102(68.9)	46(31.1)	
	Villa house	264(52.8)	202(76.5)	62(23.5)	
^a^Economic dependency	Independent	32 (64.0)	237(74.1)	83(25.9)	0.251
	Dependent	180(36.0)	140(77.8)	40(22.2)	
^a^Chronic disease	No	272(54.4)	156(68.4)	72(31.6)	**0.001**
	Yes	228(45.6)	221(81.2)	51(18.8)	
^a^Walking ability	Independent	407(81.4)	193(74.8)	65(25.2)	0.112
	Dependent	93 (18.6)	184(76)	58(24)	
^a^Use of mobility aids	No	103(20.6)	85(82.5)	18(17.5)	0.061
	Yes	397(79.4)	292(73.6)	105(26.4)	

Bold values significant at 0.05

Values are presented as number [%]

^a^Independent-sample *t*- test, ^b^one-way ANOVA

According to the results of the independent-sample *t*-test and one-way ANOVA analysis, there was a significant statistical relationship between the level of elder abuse and population variables such as the number of children, housing type, and chronic disease. Among the 500 participants, 377 (75/4%) reported experiencing at least one kind of abuse in the past year. The highest prevalence of elder abuse was observed for emotional neglect (47.2%) and psychological abuse (40.8%), while the lowest rates were measured for rejection (15.4%) and physical abuse (12.4%). The prevalence of different types of elder abuse is shown in Table 2.

**Table 2:** The frequency of domestic elder abuse subscales (*n* = 500)

Elder abuse subscales	Yes Frequency [%]	No Frequency [%]
Emotional neglect	236(47.2)	264(52.8)
Care neglect	130(26)	370(74)
Financial neglect	103(20.6)	397(79.4)
Authority deprivation	184(36.8)	316(63.2)
Psychological abuse	204(40.8)	296(52.9)
Financial abuse	176(35.2)	324(64.8)
Physical abuse	62(12.4)	438(87.6)
Rejection	77(15.4)	423(84.6)
Total abuse score	377(75.4)	123(24.6)

All data are presented as number or percent

According to the analysis of binary logistic regression in this table, it is evident that the number of children, residential status, and chronic disease are significant predictors of elder abuse. Based on the results shown in the table, an increase in the number of children (1–4 and 5) and having a chronic disease are associated with an increased risk of elder abuse. Living in an apartment is also associated with a higher risk of elder abuse (Table 3).

**Table 3:** The unadjusted binary logistic regression analysis Model of factors related to elder abuse (*n* = 500)

Variable	*p*-value	OR	95% CI	
			Lower	Upper
**Age (year)**	0.568	-	-	-
60–69	0.349	1.459	0.662	3.214
70–79	0.296	1.511	0.697	3.279
**Gender**	0.538	0.831	0.462	1.497
**Number of children**	0.054	-	-	-
0	0.098	0.272	0.058	1.272
1–4	0.035	0.565	0.332	0.959
≥ 5	0.045	5.104	1.033	25.210
**Educational level**	0.930	-	-	-
Illiterate	0.872	1.072	0.459	2.507
Primary	0.730	1.132	0.561	2.283
**Living arrangement**	0.123	-	-	-
Living with spouse	0.903	1.236	0.041	36.953
Living with spouse & children	0.859	0.735	0.025	22.027
Living with children	0.556	2.480	0.120	51.085
Living alone	0.330	4.444	0.221	89.253
**Employment status**	0.913	-	-	-
Unemployed	0.749	1.132	0.530	2.416
Employed	0.701	1.141	0.581	2.243
**Housing type**	0.048	-	-	-
Apartment	0.999	0.000	0.000	.
Home without a yard	0.999	0.000	0.000	.
Villa house	0.999	0.000	0.000	.
**Residential status**	0.212	-	-	-
Landlord	0.836	0.929	0.460	1.874
Children’s home	0.083	0.650	0.399	1.059
**Economic dependency**	0.302	-	-	-
Independent	0.262	0.208	0.013	3.241
Dependent	0.177	0.152	0.010	2.343
**Chronic disease**	0.034	1.661	1.039	2.656
**Walking ability**	0.998	-	-	-
Independent	0.999	0.000	0.000	.
Dependent	0.999	0.000	0.000	.
**Use of mobility aids**	0.863	1.131	0.278	4.601

Binary logistic regression analysis used elder abuse as the dependent variable to control confounding factors. All variables that reached a *p*-value of less than 0.05 in the independent-sample *t*-test and analysis of variance were examined as independent variables. The results showed that the number of children (1–4 reference) and chronic disease (yes concern) were significant predictors of elder abuse. Specifically, having an increased number of children (1–4) was associated with a 1.275 times higher risk of elder abuse while having a chronic illness was associated with a 0.601 times lower risk of elder abuse. The residential status and housing types did not significantly predict elder abuse (Table 4).

**Table 4:** The adjusted binary logistic regression analysis Model of factors related to elder abuse (*n* = 500)

Characteristics	*p*-value	OR	95% CI	
			Lower	Upper
Number of children (1–4 reference)	*p* < 0.001	1.275	1.137	1.430
Residential status	0.583	0.863	0.511	1.460
Housing type	0.658	0.938	0.707	1.245
Chronic disease (Yes reference)	0.020	0.601	0.391	0.922

## Discussion

In our study, we found that elder abuse was 75.4%. We also discovered that certain factors were linked to a higher severity of elder abuse, such as having a specific number of children (1–4 or 5), suffering from a chronic disease, and residing in an apartment. However, regarding the different types of elder abuse, emotional neglect had the highest prevalence at 47.2%, while physical abuse had the lowest prevalence at 12.4%.

### Elder abuse

The prevalence of elder abuse (75.4%) was significantly higher than the estimated rates in India (50.2%) [18], the China (36.2%) [19], USA (35.0%) [20] and Iran (38.5%) [21]. Other cross-sectional studies reported lower rates of elder abuse at 60.3% and 63.3%, respectively, which were lower than the rates found in our study. Regarding age, 90.4% of participants had experienced at least one form of abuse, which is higher than what was reported in our study [6, 22]. This finding completely differs from studies conducted in developed and developing countries [23]. The prevalence level we observed was also higher than that reported in the Eastern Mediterranean region, where the rates were 43.7% in Egypt [24] and 4–6% in Saudi Arabia [25]. A systematic review conducted by Dong revealed that the prevalence of elder abuse varied significantly, ranging from 2.2 to 79.7%, across five continents [26]. These variations can be attributed to cultural, social, or methodological differences. Despite having a similar definition for elder abuse, the inconsistency in results could be due to the use of different research methods, non-probability sampling, inappropriate tools, and issues related to data collection reliability. According to the disengagement theory, elderly individuals often experience social isolation and receive less attention. Their needs are sometimes neglected, even though they still can contribute [27]. In Iran, one of the main reasons for the high prevalence of elder abuse is the passive and indifferent view towards older people, which has resulted in their exclusion from natural social activities. As mentioned, the percentage of elder abuse in Ardabil was approximately twice the global average and even higher than the national average. This could be attributed to differences in lifestyle, age, gender, marital status, children’s financial status, society’s general culture, and society’s perception of older people.

### Emotional neglect and care neglect

Nearly half of all older adults in this study experienced emotional neglect. Neglect was the most prevalent form of abuse in Quddoosi’s study [28]. According to Carl Palmer et al.‘s research, the lowest rates of neglect were reported in Canada (0.4%), followed by Europe (0.5%) and the United States (1.1%) [29]. Conversely, India had the highest prevalence of neglect at 3.4% [30]. Another study in Nanjing, China, focused on the urban population revealed that 35% of older people individuals had experienced emotional abuse and neglect [31]. Christopher et al.‘s study also identified physical abuse as the second most common type of geriatric abuse after neglect [32].

In contrast to previous studies, Saatlou et al. [33] and Yang Ji Yan [34] found that emotional neglect ranked second after psychological abuse, with Mali ranking third in prevalence. According to the Disengagement theory of the older people from society, the older people is being neglected and forgotten, with their needs not being addressed. This neglect starts even before they reach the stage of imminent death when they are still capable of playing a role in society. It can lead to social and functional isolation and a lack of self-esteem [35]. The increasing busyness of work and family for children, especially in big cities and modern life, along with economic problems resulting in multiple jobs, distance, traffic, and mental fatigue, all directly and indirectly contribute to the lack of opportunity and attention given to older people.

On the other hand, there has been significant progress in the telecommunications industry in recent years. This has brought about a substantial change in communication methods. The current generation relies heavily on SMS, Telegram, Instagram, WhatsApp, etc., to communicate with others. However, most older people individuals do not have access to these facilities or possess the necessary ability to use them. Over time, this weakens communication between the older people and their children and grandchildren. As a result, emotional neglect increases.

### Financial neglect and abuse

One-third of the elder population experienced financial abuse, which was higher than the reported rates in other African countries such as Nigeria (13.3%) [36], Egypt (27%) [24], and South Africa (24.4%) [37]. Older adults who are financially stable or dependent are often targeted by disadvantaged youth due to their vulnerabilities associated with old age. These vulnerabilities include a higher risk of neurocognitive disorders and functional dependence, making it difficult for them to manage their finances or seek financial assistance when needed [6]. Additionally, they may be more susceptible to making poor financial decisions and falling victim to fraudulent schemes. The increase in functional dependency as individuals age is a significant factor that increases the likelihood of various types of abuse, excluding sexual abuse, due to their increased vulnerability and need for assistance in daily activities. Elderly individuals with a high functional dependence impose a significant care burden on others, increasing the likelihood of abuse [38]. The study’s findings confirm that financial misconduct occurs in 35.2% of cases.

### Physical abuse

The percentage of older adults experiencing physical abuse in this study (12.4%) was higher than what was reported in a systematic review of 20 studies conducted in most countries (0.2–4.9%) [39]. However, it is essential to note that the studies included in the review by Pillemer et al. (2016) measured physical abuse using a scale that primarily focused on intimate partner violence, excluding abuse from other potential perpetrators such as children, neighbors, and others – which were considered in our study [29]. This difference in measurement methods may explain the significant disparity in reported prevalence rates. On the other hand, our study’s prevalence of physical abuse was lower than reported in a Nigerian study (47.0%) [40]. This discrepancy may be attributed to the fact that other non-physical and not easily visible mistreatment may not be recognized as abusive behavior due to a lack of awareness among people. Consequently, these unrecognized forms of mistreatment may contribute to higher rates of overall misbehavior.

### Factors associated with elder abuse

Elder abuse is a particularly intricate type of domestic violence that can be affected by numerous factors.

This study found that the severity of elder abuse escalated when the number of children increased, precisely when there were either 1–4 or 5 children.

The results of this study align with the research conducted by Pengcheng Du et al. [41]. Additionally, the original survey of Aslan and Arsi found a positive correlation between the number of children and various forms of abuse and neglect towards older individuals, including physical, psychological, financial, and sexual abuse [42]. It is probable that when there are more children in a family, some may have a greater likelihood of avoiding responsibilities towards their aging parents, while those obligated to provide care become more vulnerable to objections from their spouses. Consequently, this situation leads to increased abuse towards older people parents by their children.

Our research has found that chronic illness contributes to elder abuse, which aligns with the findings of studies conducted in other countries [43, 44]. This discovery is consistent with previous research [45, 17]. Unsurprisingly, individuals with disabilities often rely on others for assistance in their daily activities [46]. Other studies have also supported these findings [43, 44]. However, Murphy did not find a direct correlation between the physical dependency of older people individuals and elder abuse. Caring for aging parents with physical disabilities and chronic illnesses requires significant support and often involves personal sacrifices from caregivers and other family members [46] .

Similarly, Wolf emphasized in his study that there is no direct link between the physical dependency of older adults and elder abuse [47]. Caring for physically disabled or ill parents requires a lot of support and can often lead to sacrificing one’s career, which can impact the well-being of family members. It can create significant stress in one’s job and affect economic, mental, and physical health. The overwhelming pressure to provide daily care for elder parents can also lead to mistreatment. Additionally, older adults with chronic illnesses and physical impairments may be unable to defend themselves or report their abusers to the police due to their vulnerability and fear of neglect.

### Limitation

This study is cross-sectional, and a prospective study should be conducted to find a causal relationship. Since the information regarding elder abuse was self-reported, it may be influenced by participants’ perceptions and biases. In the present study, the characteristics of abusers were not assessed, while mental disorders, substance abuse, and alcohol consumption may increase the risk of elder abuse. Lastly, the current research utilized an Iranian native tool and even considered a specific type of harassment and distress as elder abuse in the past year. Therefore, the prevalence of elder abuse may be higher than the current situation.

## Conclusion

This study reported a relatively high prevalence of elder abuse, with 75.4% of the 500 participants reporting experiencing at least one type of abuse in the past year. It was also found that the number of children, housing type, and chronic disease were significant predictors of elder abuse. Based on the results of this study, it is suggested that educational and awareness programs be implemented in the community to reduce the prevalence of elder abuse. These programs can include providing information about the causes and consequences of elder abuse, how to report elder abuse, and how to prevent it. Additionally, proactive measures such as creating social networks for older adults, developing supportive and counseling services, and strengthening legal control can reduce elder abuse’s prevalence. Finally, access to accurate information and statistics on elder abuse should be improved to increase awareness and transparency in reporting such cases.

## Data Availability

The datasets used and/or analyzed during the current study are available from the corresponding author on reasonable request.
